# Hypnotisability and the Cerebellum: Hypotheses and Perspectives

**DOI:** 10.1007/s12311-021-01292-1

**Published:** 2021-10-15

**Authors:** Enrica Laura Santarcangelo, Diego Manzoni

**Affiliations:** Department of Translational Research and New Technologies in Medicine and Surgery, Laboratory of Cognitive and Behavioural NeuroscienceVia San Zeno, University of Pisa, 31, 56127 Pisa, Italy

**Keywords:** Motor cortex, Sensorimotor, Imagery, Pain, Attention, Behaviour

## Abstract

Hypnotisability is a multidimensional trait predicting the proneness to enter hypnosis and/or accept suggestions and is associated with several psychophysiological correlates. This scoping review reports the differences between individuals with high (highs) and low hypnotizability (lows) in the left cerebellar lobules IV–VI grey matter volume, in the excitability of the right motor cortex and in motor and non-motor functions in which the cerebellum may be involved. A reduced cerebellar inhibition may explain the greater excitability of the highs’ right motor cortex. The latter may be involved in their greater proneness to ideomotor behaviour following sensorimotor suggestions. The associated experience of involuntariness and effortlessness could be due to the motor cortex greater excitability as well as to activation of a specific cerebellar-parietal circuit. Looser postural and visuomotor control with no learning across trials and greater attentional stability can be accounted for by a less accurate cerebellar predictive model of information processing. The highs’ stronger functional equivalence between imagery and perception/action and greater motor excitability may be involved in the highs’ greater proneness to respond to emotional stimuli. Paradoxical pain control may depend on reduced cortical inhibition of the pain matrix by the cerebellum. Cerebellar hypotheses are not alternative to other physiological mechanisms and should be tested in future research.

Hypnotisability is a multidimensional trait measured by standard scales. It predicts the proneness to enter hypnosis and to accept suggestions aimed at modifying perception, memory and behaviour also in the ordinary state of consciousness. It is associated with several physiological, cognitive and emotional correlates [5] suggesting a bio-psycho-social construct [7].

Brain morpho-functional studies have been focused on the differences between highly (highs) and low hypnotizable individuals (lows) in the activity and connectivity of the default mode, salience and executive circuits (Landry et al., 2017). The possible cerebellar differences have been largely disregarded, with the sole exception of the report of reduced grey matter volume in the left lobules IV–VI of highs with respect to lows [12]. Here, we present a few findings pointing to hypnotizability-related differences potentially related to cerebellar functions. As a motor structure, in fact, the cerebellum is involved in sensorimotor integration and motor learning, preparation and control, and as a non-motor structure, it participates in several executive processes ranging from attentional control to social cognition (Hull et al., 2020).

The reported findings have been obtained in not hypnotized participants.

## Motor Cortex Excitability and Functional Equivalence Between Imagery and Perception/Action

Hypnotizability is positively correlated with the excitability of the right motor cortex in resting conditions and during sensori-motor imagery [14]. The reduced grey matter volume (GMV) of the left cerebellar lobules IV–V [12], which exert a tonic inhibition of the contralateral motor cortex through the dentate nucleus, could be responsible for this finding. Neuropsychological tests and studies of the catechol-O-methyl-transferase polymorphism, however, suggest that higher dopaminergic tone could also be involved the highs’ greater motor cortex excitability. In this case, the dopaminergic effects should not be limited to motor areas, but there is no evidence of a generalized greater cortical excitability in highs. No significant difference between highs and lows in the noradrenergic supply to the cerebral cortex by the locus coeruleus is involved [8].

Greater excitability of the motor cortex can account for the highs’ stronger functional equivalence (FE) between sensori-motor imagery and perception/action. Stronger FE may be responsible for the highs’ greater proneness to ideomotor behaviour and for the involuntariness and effortless of action they report after movement induced by sensori-motor suggestions (Ibanez-Marcelo et al., 2019). Reduced sense of agency has also been reported for movements induced by suggestions during hypnosis and has been attributed to activation of a cerebellar-parietal circuit [1]. The cerebellum may play a key role in both facts.

## Sensori-Motor Integration

Suppression of vision and alteration of the legs proprioceptive information induce larger and faster body sway in standing highs than in lows, despite the similar perception of sway. Stabilogram diffusional analysis reveals a set point difference, since in highs, the position of the centre of pressure is corrected by sensory re-afferences for larger sway amplitudes than in lows. In addition, no learning across successive trials reducing body sway occurs in highs, in contrast to lows. Moreover, keeping the head rotated toward one side (which changes the vestibular information coding) increases the velocity of sway only in lows [13].

In a visuomotor task – launching small balls toward a target – highs are less precise than lows. They exhibit larger variability and no learning across consecutive trials. When the direction of gaze is deviated by prisms, however, both highs and lows change the direction of launches as occurs in the general healthy population, but highs still exhibit larger errors and variability than lows, and absence of learning across trials [13].

In a task of proprioceptive identification of far and near hand positions reached at the end of active and passive movements in the horizontal plane, lows display the same accuracy after both movements, whereas highs exhibit greater accuracy after the active than passive movements. This suggests that central commands are more relevant to the highs’ than to the lows’ performance, possibly due to less efficient integration of peripheral information. In the same vein, the lows’ accuracy is larger than highs for the large distances from the starting position, which again suggests that lows can rely on peripheral information better than highs [10].

A less accurate predictive model of sensorimotor integration by the cerebellum may account for the highs’ reported characteristics, as the cerebellum prepares neural systems for signal acquisition, analysis, and for the reaction to them (Hull et al., 2020).

Lower spontaneous blink rate has been observed in highs with respect to mediums and lows in the earliest minutes of a relaxation session [13]. This could be accounted for by reduced cerebellar control of the blinking circuit due to the reduced grey matter volume of the left lobule VI [3], although, not alternatively, also by higher dopaminergic tone. In both cases, the interaction between the default mode network and the executive circuit (Landry et al., 2017) could be responsible for the highs’ reduction of blink rate after a few minutes of relaxation.

## Cognitive Performance

The cerebellar connections to the prefrontal and parietal cortex and the association between cerebellar peculiarities and personality traits (Petrosini et al., 2017) suggest that a few cognitive aspects of hypnotizability may be at least partially influenced by the cerebellum.

Highs display greater attentional stability allowing them to maintain the focus of attention (“absorption”) making its re-direction difficult [5]. Since the cerebellum processes cognitive information similarly to sensorimotor signals, according to the predictive model of information processing (Hull et al., 2020), the highs’ attentional stability/absorption could represent the non-motor aspect of their “inertia” in sensori-motor integration. Not alternatively, however, greater absorption can be attributed to higher dopaminergic tone [13].

A paradoxical pain control has been observed in highs undergoing nociceptive stimulation of the left hand after transcranial bilateral anodal cerebellar stimulation (tDCS). In fact, they do not report a significantly reduced pain intensity and show larger rather than smaller ERPs amplitudes, in contrast to the general population. We hypothesize that the highs’ cerebellar cortex has a poor inhibitory effect on cerebellar nuclei and, thus, an excitatory rather than inhibitory effect on the pain matrix (Bocci et al., 2018) whose activation is not balanced by the activation of descending analgesic pathways by the motor cortex (Table 1).

**Table 1:** Characteristics of highly hypnotizable individuals possibly dependent on cerebellar peculiarities

**experimental findings**	**hypotheses**
greater excitability of the right motor cortex	reduced cerebral cortical inhibition by the left cerebellar lobules IV-V
*Spina et al., 2020*	*higher cortical dopaminergic tone*
greater functional equivalence (FE) between sensorimotor imagery and perception/action	greater excitability of the motor cortex due to reduced cerebellar inhibition
*Ibanez-Marcelo et al., 2019*	*higher dopaminergic tone*
looser postural and visuomotor control,no learning effects	less accurate cerebellar predictive model of sensory/motor integration
*Santarcangelo & Scattina, 2016*	
better proprioceptive identification of hand positions after active than passive movements	major role of central commands in the cerebellar sensori-motor integration
*Padilla -Castaneda et al., 2015*	due to less accurate cerebellar predictive model of sensory/motor integration
higher blink rate	reduced cerebellar inhibition of the occipital cortex
*Santarcangelo & Scattina, 2016*	*higher cortical dopaminergic tone*
greater attentional stability/absorption	less accurate cerebellar predictive model of information processing
*Lanatà et al., 2020 *	*higher cortical dopaminergic tone*
paradoxical pain experience after cerebellar anodal stimulation	reduced inhibition of the pain matrix by the cerebellar lobule VI
*Bocci et al., 2018*	
higher activity of the Behavioral Approaching System	reduced cerebellar inhibition of motor areas
*Diolaiuti et al., 2019*	reward anticipation through stronger FE

Note. Some findings are interpreted as indirect effects of cerebellar peculiarities (for instance, stronger FE caused by greater excitability of motor areas depending on reduced cerebellar inhibition and/or higher dopaminergic tone). Alternative hypotheses (*Italics)* are not mutually exclusive.

The activity of the behavioural approach system (BAS), related to motivational and reward-oriented behaviour, is higher in highs than in mediums and lows. The highs’ stronger functional equivalence between imagery and perception could allow them to anticipate the experience of reward through imagery (Ibanez-Marcelo et al., 2019), making them more prone to goal-directed actions. In contrast, the lower proneness of both highs and lows with respect to mediums to withdrawal from possibly unpleasant situations (behavioural inhibition system, BIS) could be sustained by different mechanisms possibly related to the interactions of the limbic regions involved in BAS and BIS with several cerebral regions [4].

## Conclusions and Future Perspectives

The cerebellum may play a relevant role in hypnotizability-related physiology and behaviour. A cerebellar less accurate predictive model of information processing (Hull et al., 2020) may account for most of the findings of sensori-motor integration and, at least partially, attentional stability (Santarcangelo and Sebastiani, 2016). Increased excitability of the highs’ right motor area [14] can account for the highs’ stronger functional equivalence between imagery and perception/action, thus for their proneness to ideomotor behaviour which is experienced as effortless and involuntary (Ibanez-Marcelo et al., 2019). This subjective experience could be also due to greater excitability of the motor cortex making action more likely as well as, during hypnosis, to a reduced sense of agency associated with activation of a cerebellar-parietal circuit. The highs’ greater proneness to respond to a few emotional stimuli [4] could also be related to the excitability of the motor cortex, as well as to greater disposition toward anticipation of reward through imagery (Ibanez-Marcelo et al., 2019). The hypnotizability-related mode of sensorimotor integration and emotional traits could be relevant to the interpretation of patients’ behaviours, the highs’ stronger of FE could be used as a predictor of better outcomes in neurological patients submitted to brain computer interface.

All these hypotheses should be experimentally tested in future research enrolling also medium hypnotizable participants, who represent most of the population. The role of co-operative and/or alternative mechanisms possibly involved in the described hypnotizability-related features should be clarified.

Work in progress will ascertain whether the brain availability of endothelial nitric oxide (NO) is larger in highs than in lows, as occurs for the brachial artery during mental stress and nociceptive stimulation. In physiological conditions, in fact, NO exerts a positive influence on the brain development, maturation and plasticity, whereas excessive/uncontrolled NO availability could impair the maturation of the nervous tissue and particularly of granule cells, which are the most sensitive to NO toxicity [13]. This may play a role in the highs’ cerebellar morpho-functional characteristics.
